# Annotating regulatory elements by heterogeneous network embedding

**DOI:** 10.1093/bioinformatics/btac185

**Published:** 2022-03-24

**Authors:** Yurun Lu, Zhanying Feng, Songmao Zhang, Yong Wang

**Affiliations:** CEMS, NCMIS, HCMS, MADIS, Academy of Mathematics and Systems Science, Chinese Academy of Sciences, Beijing 100190, China; School of Mathematics, University of Chinese Academy of Sciences, Chinese Academy of Sciences, Beijing 100049, China; CEMS, NCMIS, HCMS, MADIS, Academy of Mathematics and Systems Science, Chinese Academy of Sciences, Beijing 100190, China; School of Mathematics, University of Chinese Academy of Sciences, Chinese Academy of Sciences, Beijing 100049, China; CEMS, NCMIS, HCMS, MADIS, Academy of Mathematics and Systems Science, Chinese Academy of Sciences, Beijing 100190, China; CEMS, NCMIS, HCMS, MADIS, Academy of Mathematics and Systems Science, Chinese Academy of Sciences, Beijing 100190, China; School of Mathematics, University of Chinese Academy of Sciences, Chinese Academy of Sciences, Beijing 100049, China; Center for Excellence in Animal Evolution and Genetics, Chinese Academy of Sciences, Kunming 650223, China; Key Laboratory of Systems Biology, Hangzhou Institute for Advanced Study, University of Chinese Academy of Sciences, Chinese Academy of Sciences, Hangzhou 330106, China

## Abstract

**Motivation:**

Regulatory elements (REs), such as enhancers and promoters, are known as regulatory sequences functional in a heterogeneous regulatory network to control gene expression by recruiting transcription regulators and carrying genetic variants in a context specific way. Annotating those REs relies on costly and labor-intensive next-generation sequencing and RNA-guided editing technologies in many cellular contexts.

**Results:**

We propose a systematic Gene Ontology Annotation method for Regulatory Elements (RE-GOA) by leveraging the powerful word embedding in natural language processing. We first assemble a heterogeneous network by integrating context specific regulations, protein–protein interactions and gene ontology (GO) terms. Then we perform network embedding and associate regulatory elements with GO terms by assessing their similarity in a low dimensional vector space. With three applications, we show that RE-GOA outperforms existing methods in annotating TFs’ binding sites from ChIP-seq data, in functional enrichment analysis of differentially accessible peaks from ATAC-seq data, and in revealing genetic correlation among phenotypes from their GWAS summary statistics data.

**Availability and implementation:**

The source code and the systematic RE annotation for human and mouse are available at https://github.com/AMSSwanglab/RE-GOA.

**Supplementary information:**

Supplementary data are available at *Bioinformatics* online.

## 1 Introduction

Ontology building and ontology annotation are fundamental in biology (Thomas *et al.*, 2007). A large number of function prediction and annotation methods have been proposed in the past decades, either at pathway level from the point of view of biochemical reactions or at gene level by its regulatory element (RE) and its protein product (Zhou *et al.*, 2019). With the development of high-throughput experiment technologies, REs located in the 98% non-coding regions of genome have been intensively studied for their context specific regulatory functions from different aspects. For instance, Nord *et al.* (2020) discussed neurobiological functions of transcriptional enhancers; Field *et al.* (2020) studied how enhancers regulate their target genes and how enhancers and promoters communicate; Fishilevich *et al.* (2017) provided an online resource for enhancer–gene relations; and Li *et al.* (2020) described CRISPR/dCas9-based enhancer-targeting epigenetic editing systems, enCRISPRa and enCRISPRi, for efficient analysis of enhancer functions *in situ* and *in vivo*. These works highlighted the necessity and feasibility to apply ontology analysis at the regulatory element resolution. Meanwhile, this task is either costly or tailor-made, and systematical and computational methods are in pressing need.

A widely adopted strategy for interpreting RE’s function is to borrow annotations from its ‘nearest gene’. GREAT (McLean *et al.*, 2010) associated REs with genes based on a regulatory domain that extends in both directions to the nearest gene's Transcription Start Site (TSS). Such methods ignored the complex many to many RE-gene mapping in the gene regulation. On one hand, *cis*-regulatory elements can control a gene far away in genome (Ribich *et al.*, 2006) instead of solely regulating the nearest genes, or regulate multiple genes (Zhang *et al.*, 2019). This difficulty was emphasized by a recent study revealing that non-coding REs associated with a human craniofacial disorder causally affect two clusters of enhancers regulating SOX9 expression during a restricted window of facial progenitor development at a distance up to 1.45 Mb (Ribich *et al.*, 2006). Those REs are located far away outside of genes and fail the routine way to annotate non-coding regions with genes located within 500 kb, such as GREAT (McLean *et al.*, 2010). In addition, this dramatic case points out that RE’s function should be studied in specific cellular contexts. On the other hand, some target genes of *cis*-regulatory elements are translated to transcription factors (TF) and then regulate other genes. Therefore, the relationships among REs and genes are better represented as a network containing more than one type of nodes and edges due to the complexity of gene regulation. The topological structure of the whole network rather than the local neighbor genes allows a better understanding of REs’ functions.

We recently proposed a Paired Expression and Chromatin Accessibility analysis (PECA) method to reconstruct context-specific regulatory network (Duren *et al.*, 2019). The Gene Regulatory Network (GRN) constructed by PECA consists of four types of nodes: RE, TF, chromatin regulator (CR) and target gene (TG), and three types of edges: CR recruitment to RE, TF binding to RE and RE regulating TG. Our heterogeneous network introduces REs as harbors to link diverse molecules to determine gene expression level, which extends traditional regulatory networks that only describe the interactions among TFs and their target genes. This is a valuable asset for functional annotation of REs in a context specific regulation process (Heinz *et al.*, 2015). Meanwhile, powerful network embedding and graph neural network approaches have been recently developed and hold the promise to effectively integrate diverse types of information (Sharan *et al.*, 2007,  Shi *et al*., 2017). For example, metapath2vec (Dong *et al.*, 2017) can embed a heterogeneous network into vector representations and is successfully used by GEEK (Cao *et al.*, 2020) for an integrative study of heterogeneous gene regulatory mechanisms. Another network embedding application is the transferred multi-relational embedding model Bio-JOIE (Hao *et al.*, 2020), which captures the knowledge of Gene Ontology (GO) and Protein-Protein Interaction (PPI) networks and demonstrates its superb capability in modelling the SARS-CoV-2-human protein interactions.

In this article, we propose a heterogeneous network embedding-based approach for annotating REs with GO terms by integrating Gene Ontology Annotation (GOA) with two biological networks including PPI and GRN. GOA can describe the features of genes and gene products from different aspects (Ashburner *et al.*, 2000), PPIs present physical contacts between proteins in the cell; GRN models the regulatory relations among TFs, REs and genes. Together, a framework for RE functional annotation, Regulatory Elements Gene Ontology Annotation (RE-GOA), integrates heterogeneous biological networks and generates resources of REs annotation for human and mouse. To demonstrate the utility of RE-GOA, we apply it to analyzing three different types of data including TF binding sites from ChIP-seq data, differential accessible peaks from ATAC-seq and GWAS summary statistics. The results show that RE-GOA is a promising tool for annotation of regulatory elements.

## 2 Materials and methods

### 2.1 Overview of Gene Ontology Annotation method for Regulatory Elements (RE-GOA)

**Fig. 1.**
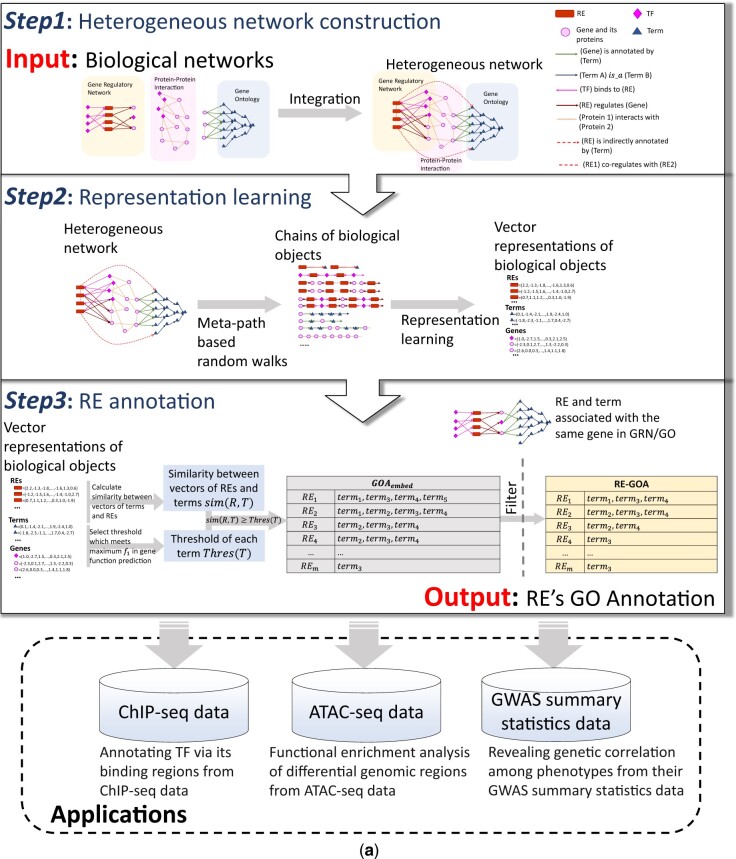
Schematic diagram of RE-GOA framework. RE-GOA takes three biological networks including GRN, PPI, GOA as input and REs’ annotation as output. The three major steps include: (1) constructing heterogeneous network; (2) network embedding; (3) associating REs and GO terms. To demonstrate the high quality of RE-GOA, three applications are carried out including annotating TF via its binding regions from ChIP-seq data, functional enrichment analysis of differential genomic regions from ATAC-seq data, and revealing genetic correlation among phenotypes from their GWAS summary statistics data

We propose a network embedding-based framework, RE-GOA, for annotating regulatory elements. As shown in Figure 1, RE-GOA takes three biological networks including GOA, PPI and GRN as input, utilizes the global and local topological information from all these networks, and outputs functional annotations to REs. The three networks, GOA, PPI and GRN, contain different information of genes and other biological objects. GO terms can be divided into three parts to respectively describe molecular functions (MF) that genes and gene products have, biological processes (BP) accomplished by multiple molecular activities, and the locations relative to cellular components (CC) where genes and gene products perform functions. GO terms are structured as a Directed Acyclic Graph (DAG) and used to annotate genes and gene products in GO Annotation. PPIs are abstracted as a weighed undirected network, where proteins are represented as nodes and the weight of edges represents the interaction strength between proteins. GRN describes weighted directed regulations among genes, regulatory elements and transcription factors in certain cellular contexts. It was reconstructed from paired expression and chromatin accessibility data (Duren *et al.*, 2019).

As shown in Figure 1, RE-GOA annotates REs with GO terms by three steps. First, biological networks including GRN, PPI and GOA are integrated into a heterogeneous network with four types of nodes: GO terms, genes, TFs and REs, and five types of edges: term A is_a term B, gene is annotated by term, protein (gene) C interacts with protein (gene) D, TF binds to RE and RE regulates gene. In addition, two relationships can be inferred from the above existing edges. An RE is indirectly annotated by a term if one of RE’s target genes is annotated by the term. Two REs co-regulate if they regulate the same gene.

**Fig. 2.**
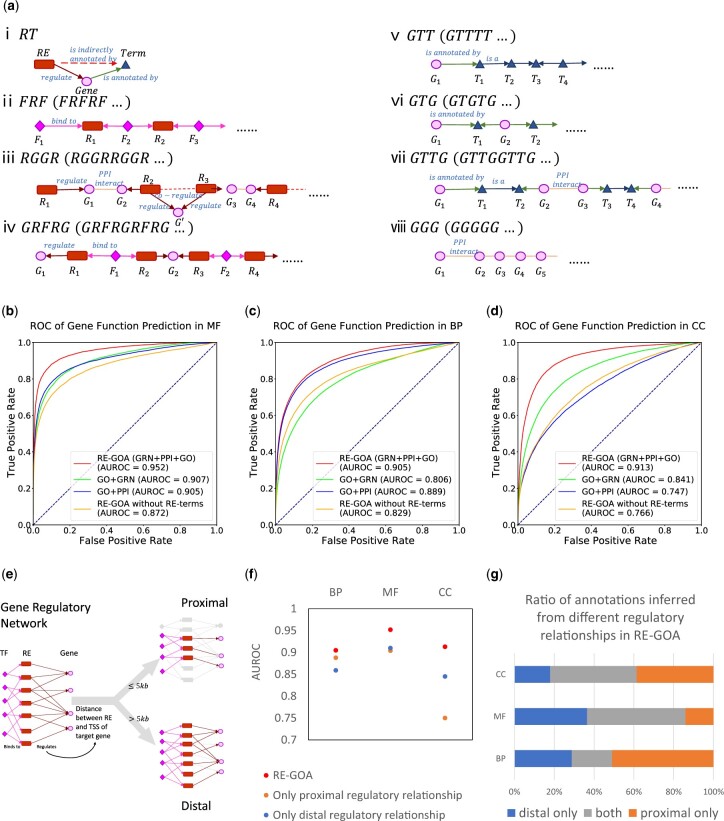
RE-GOA captures the global and local structure from the heterogeneous biological networks and can accurately annotate function. (**a**) Illustration of the meta-paths defined for representation learning to capture the diverse information in the heterogeneous network. Each meta-path is an ordered sequence of node types representing that two nodes are connected with a relationship. Random walks are generated based on the meta-paths and used for representation learning. (**b–d**) Integration of the networks outperforms using only part of the meta-paths in gene function prediction. Integrating three networks (GOA + PPI + GRN) and embedding with defined meta-paths result in higher AUROC in gene function prediction than using only two of them (GOA + PPI and GOA + GRN). The inferred, indirect annotation relations between REs and terms also improve accuracy. (**e**) Illustration of the strategy to divide GRN into two parts: Proximal (distance of REs to its target gene within 5 kb) and Distal (distance larger than 5 kb). (**f**) The proximal and distal parts of GRN both play an important role in RE-GOA. Discarding either of the two parts reduces the precision of gene function prediction. (**g**) Ratio of annotations inferred from distal, proximal or both regulatory relationships of REs in RE-GOA. Taking advantage of both distal and proximal infers over 50% of the annotations in RE-GOA

Second, four types of nodes in the constructed heterogeneous network can be embedded into vector space by representation learning. The key idea behind this step comes from Natural Language Processing (NLP), where the goal is to learn an embedding for each node such that the resulting node vectors can naturally capture their neighborhood in the network. Technically, this is done by meta-path-guided random walks in the heterogeneous network followed by a word2vec as a one-layer artificial neural network called skip-gram (Mikolov *et al.*, 2013a,b). Eight different meta-paths (Fig. 2a) are defined according to the domain knowledge on gene regulation and can capture useful information in the network.

Once the GO terms, REs and genes in the heterogeneous network are embedded into a low-dimensional vector space, we can systematically assign terms to REs as the third step. A GO term whose embedding vector has larger cosine similarity with an RE indicates a higher possibility of existence of a latent relationship between the term and RE (Fig. 1, Step 3). To battle the complex hierarchical relationships in GO, we determine $\mathrm{Thres}\left(T\right)$ based on cosine similarity as the threshold of term T to achieve maximum $f_{1}\;\mathrm{value}$ using gene function annotation as gold standard. By checking the GO terms of closely related target genes for REs, RE-GOA is finally produced by filtering out terms lower than their thresholds. The detailed workflow is shown in Supplementary Figure S1.

### 2.2 Constructing a heterogeneous network

The GO hierarchy and GOA, GRN and PPI networks contain different information for biological objects. Here we integrate these networks into a heterogeneous network with four types of nodes: GO terms, genes (or proteins), TFs and REs (the CR nodes in GRN are ignored), and seven types of edges where five of them are obtained directly from original networks, and two types of edges inferred from existing edges:

1. term $T_{1}\;is_{a}$ term $T_{2}$ obtained from Gene Ontology hierarchy, where ‘$is_{a}$’ means that term $T_{1}$ is a child node of term $T_{2}$ in GO;
2. gene G being annotated by a term T obtained from GOA which is downloaded from AmiGO (http://amigo.geneontology.org/amigo/search/annotation), and adjustments are made according to the True Path Rule (Blake 2013), declaring that if a gene is annotated by a GO term t, then it is also annotated by t’s ancestor terms. So here we annotate genes with terms annotating them in AmiGO and their ancestors in GO, which means:
  $$\mathrm{GO}A_{g}\left(G\right)=\cup_{t\in \mathrm{GO}A_{\mathrm{Ami}}\left(G\right)}\;\left\{t^{\prime }|t^{\prime }is\;\mathrm{an}\;\mathrm{ancestor}\;\mathrm{of}\;t\right\}\cup \;\mathrm{GO}A_{\mathrm{Ami}}\left(G\right),$$
  where $\mathrm{GO}A_{g}\left(G\right)$ represents the set of terms annotating G in our method and $\mathrm{GO}A_{\mathrm{Ami}}\left(G\right)$ is the raw annotation set of gene G in AmiGO;
3. protein (gene) $G_{1}$ interacting with protein (gene) $G_{2}$obtained from PPI network;
4. TF F binding to RE R obtained from GRN;
5. RE R regulating gene G obtained from GRN;
6. RE R being indirectly annotated by term T if there exists a gene G such that R regulates G and G is annotated by T;
7. RE $R_{1}$coregulating with RE $R_{2}$ if there exists a gene G such that $R_{1}$ regulates G and $R_{2}$ regulates G.

The following rules are defined for inferring indirect edges:

R regulates G;G is annotated by T⇒R is indirectly annotated by T,

 

$$R_{1}\;\mathrm{regulates}\;G;R_{2}\;\mathrm{regulates}\;G\Rightarrow R_{1}\;\mathrm{coregulates}\;\mathrm{with}\;R_{2},$$

For convenience, we define

$$\mathrm{GO}A_{\mathrm{raw}}\left(R\right)=\cup_{G\in \mathrm{reg}\left(R\right)}GOA_{g}\left(G\right)$$

where $\mathrm{reg}\left(R\right)$ is the set of genes which RE R regulates in GRN, and $\mathrm{GO}A_{\mathrm{raw}}$ stores the indirect annotations to REs inferred from GRN and GOA.

### 2.3 Embedding the constructed heterogeneous network

Biological networks are heterogeneous with different types of nodes and edges, and embedding methods could flexibly integrate these heterogeneous data and provide a low-dimensional representation of the data for downstream tasks. Taking the constructed heterogeneous network as input, we convert the information contained in the network structure into embedding vectors in a low-dimensional space, such that the vector of each node in this space form a signature of the node that captures hidden associations in the network.

Metapath2vec provides an effective way for representation learning of heterogeneous network (Dong *et al.*, 2017), which formalizes meta-paths-based random walks to construct the heterogeneous neighborhood of a node and then leverages a heterogeneous skip-gram model to perform node embedding. A heterogeneous network is defined as a graph $G=\left(V,E,T\right)$, in which V and E are sets of nodes and edges, and $T=\left\{T_{V},T_{E}\right\}$. Each node v and edge e are associated their mapping functions $\phi \left(v\right):V\to T_{V}$ and $\varphi \left(e\right):E\to T_{E}$, respectively, indicating their types, where $T_{V}$ and $T_{E}$ denotes the set of node and edge types ($|T_{V}\;|+|T_{E}\;|>2$) (Pal *et al.*, 2016). Given a heterogeneous network $G=\left(V,E,T\right)$, metapath2vec defines the objective function to maximize the probability of the heterogeneous context $N_{t}\left(v\right)$, $t\in T_{V}$ given a node v as follows (Dong *et al.*, 2017).

$$\arg\underset{\theta }{\max}\Sigma_{v\in V}\Sigma_{t\in T_{V}}\Sigma_{c_{t}\in N_{t}\left(v\right)}\log p\left(c_{t}|v;\theta \right)\;$$

where $N_{t}(v)$ denotes v's neighbors with the $t^{th}$ type of nodes, $p(c_{t}|v;\theta )=\frac{e^{X_{c_{t}}\cdot X_{v}}}{\Sigma_{u\in V}\;e^{X_{u}\cdot X_{v}}\;}$, where $X_{v}$ is the embedding vector for node v, and $\theta =\{v\to X_{v}\in R^{d}\;|v\in V\}$ denotes embedding vectors of the nodes.

Combining meta-paths-guided random walk and representation learning allows us to integrate the highly heterogeneous data within a single general framework. The simple form of the meta-paths also allows the utility of different network components separately. To capture network information relevant to RE’s function, meta-paths are required as input for encoding domain knowledge. Each meta-path is an ordered sequence of node types connecting biological objects with one or more relationships. For example, when G representing a gene and R representing an RE, the meta-path ‘RGGR’ connects two REs ($R_{1}$ and $R_{2}$) indirectly through gene $G_{1}$ regulated by RE $R_{1}$, gene $G_{2}$ interacting with gene $G_{1}$, and RE $R_{2}$ regulating gene $G_{2}$. Here for convenience, we use ‘T’ to stand for a GO term, ‘G’ a gene, ‘R’ an RE and ‘F’ a TF. Fully considering the potential relationships of the biological objects involved in RE’s biological functions, we define eight meta-paths as follows which are also shown in Figure 2a.

-
RT , which connects RE with the terms annotating it, capturing local information transformed from gene to RE that regulates the gene;
-
FRF (FRFRF…), which connects RE with the TF having the ‘binding_to’ relationship in GRN, capturing the relationship between TF and RE, under the assumption that REs binding with the same TF tend to have similar function;
-
RGGR (RGGRRGGR…), which connects two REs if they regulate the same gene in GRN, connects two genes whose protein products have interactions in the PPI network, and connects RE and gene if they have the ‘regulate’ relationship in GRN;
-
GRFRG (GRFRGRFRG…), which connects gene and RE if they have the ‘regulate’ relationship in GRN, and connects RE with TF which have the ‘binding_to’ relationship in GRN. The two meta-paths GRFRG and RGGR connect RE with the genes it regulates, and the REs co-regulate with it, capturing global information transferred in the heterogeneous network;
-
GTT (GTTT…), which connects G with the terms annotating it, and connects two terms if they have the ‘is_a’ relationship in GO hierarchy;
-
GTG (GTGTGT…), which connects G with the terms annotating it. The meta-paths GTT and GTG , respectively, capture relations between genes and GO terms and relations among GO terms;
-
GTTG (GTTGGTTG…), which connects G with the terms annotating it, connects two terms if they have the ‘is_a’ relationship in GO, and connects two genes whose protein products have interactions in the PPI network;
-
GGG (GGGGG…), which connects two genes whose protein products have interactions in the PPI network. Meta-paths GTTG and GGG capture information from PPI, assuming that genes interacting with each other share similar function.

Guided by the meta-paths defined, we conduct random walks in the heterogeneous network. Those random walks are then formed into a corpus where the nodes are taken as words and walks as sentences. We use the word2vec package in python with skip-gram model (Mikolov *et al.*, 2013a,b) to train the corpus and obtain an embedding of the heterogeneous network which represents nodes with vectors.

In the subsequent sections, we use v(T), v(G), v(R) and v(F) to denote the embedding vectors of GO terms, genes, REs and TFs, respectively. The whole algorithm of the heterogeneous network embedding is described in Supplementary Algorithm S1.

### 2.4 Assigning GO terms to REs

We can compute the biological objects’ pairwise cosine distance based on their embedding vectors. Nodes tend to have vectors with larger cosine similarity if they are more adjacent in the walks generated according to the defined meta-paths. So, we assign a GO term to an RE or gene when their embedding vectors have larger enough cosine similarity.

GO terms are located in different levels in hierarchies of the ontology and provide different amount of information. The deeper the terms are located in hierarchies, the more concrete information they provide. With various features, terms have different similarities with genes. Thus, a big challenge is to determine a different, specific threshold for each term to annotate REs. In this section, we will annotate REs in three steps: first calculate threshold for each term, then generate $GOA_{\mathrm{embed}}$ which annotates REs with terms according to the thresholds and embeddings, and lastly, RE-GOA is finally produced by intersect $GOA_{\mathrm{embed}}$ and $GOA_{\mathrm{raw}}$.

#### 2.4.1 Calculating threshold for each GO term

Determining suitable threshold of vectors’ cosine similarity for each term is very important for getting proper annotation of REs. As assumed, a GO term tends to annotate a gene when they have similar vector representations. So, we use gene function prediction task to calculate threshold for each term. First of all, we define similarity between terms and genes as follows according to the True Path Rule.

$$\mathrm{sim}(G,T)=\underset{T^{\prime }\in \mathrm{des}\left(T\right)\cup \left\{T\right\}}{\max}\mathrm{cossim}(v(G),v(T'))$$

where des(T) denotes the descendants of term T in GO hierarchies.

More stringent thresholds with high precision and low recall may cause fewer annotations for each RE, and looser thresholds with low precision and high recall may draw in more mistakes. Here, we define Thres(T) as the threshold with which the term can get maximum $f_{1}\;\mathrm{value}\;$in gene function prediction task.

$$\mathrm{Thres}(T)=\arg\underset{t\in \left(-1,1\right)}{\max}\frac{2pre_{t}\left(T\right)*\mathrm{re}c_{t}\left(T\right)}{\mathrm{pr}e_{t}\left(T\right)+\mathrm{re}c_{t}\left(T\right)}$$

where $\mathrm{pr}e_{t}\left(T\right)=\frac{\left|G_{\mathrm{standard}}\left(T\right)\cap \;G_{\mathrm{predict}}\left(T,t\right)\right|}{\left|G_{\mathrm{predict}}\left(T,t\right)\right|}$,$\;\mathrm{re}c_{t}\left(T\right)=\frac{\left|G_{\mathrm{standard}}\left(T\right)\cap \;G_{\mathrm{predict}}\left(T,t\right)\right|}{\left|G_{\mathrm{standard}}\left(T\right)\right|}$, in which $G_{\mathrm{standard}}\;(T)=\{G|T\in \mathrm{GO}A_{g}(G)\}$ and $G_{\mathrm{predict}}(T,t)=\{G|\mathrm{sim}(G,T)>t\}$.

We only consider terms that are associated with genes and define threshold specific to each term. Such thresholds can make a balance between precision and recall. The detailed algorithm for threshold determination is described in Supplementary Algorithm S2.

#### 2.4.2 Annotating RE with GO terms

After calculating the threshold for each term, we annotate RE with terms according to the thresholds and embeddings. Specifically, we calculate similarity between REs and terms first as follows:

$$\mathrm{sim}(R,T)=\underset{T^{\prime }\in \mathrm{des}\left(T\right)\cup \left\{T\right\}}{\max}\mathrm{cossim}(v(R),v(T\prime ))$$

We define $\mathrm{GO}A_{\mathrm{embed}}(R)$ as the terms whose similarity with RE R is larger than its threshold as follows:

$$\mathrm{GO}A_{\mathrm{embed}}\left(R\right)=\cup_{T\in \mathrm{Anno}\left(R\right)}\;\mathrm{anc}(T)\cup \mathrm{Anno}(R)$$

where Anno(R)={T|sim(R,T)>Thres(T)} and anc(T) denotes the ancestors of term T.

With $GOA_{\mathrm{embed}}$ and $\mathrm{GO}A_{\mathrm{raw}}$, we now annotate REs with their intersection, i.e. terms in both $\mathrm{GO}A_{\mathrm{raw}}$ and $\mathrm{GO}A_{\mathrm{embed}}$:

$$\mathrm{GO}A_{\mathrm{re}}(R)=\mathrm{GO}A_{\mathrm{embed}}(R)\cap \;\mathrm{GO}A_{\mathrm{raw}}(R)$$

The complete annotation algorithm is described in Supplementary Algorithm S3.

### 2.5 Assembling biological network data

Gene Ontology, structured as a DAG, is a foundation for computational analysis of large-scale molecular biology and genetics experiments in biomedical research. Terms in Gene Ontology are divided into three non-overlapping ontologies, Molecular Function (MF), Biological Process (BP) and Cellular Component (CC). Terms in GO and is_a relations between terms are extracted in our studies to construct a DAG, whose nodes represent the terms and edges represent the is_a relations between terms. ‘$T_{\mathrm{child}}\;is_{a}T_{\mathrm{parent}}$’ means $T_{\mathrm{child}}$ specializes $T_{\mathrm{parent}}$ in GO, and a term may have more than one parent term. GO annotations associate genes and gene products with terms to describe a gene’s function in molecular level, the cellular components where it functions, and the biological processes it participates in. The Gene Ontology and GO Annotation are available at http://geneontology.org/.

Protein–Protein Interaction (PPI) networks are mathematical representations of the physical contacts between proteins in the cell. These contacts occurring between defined binding regions in the proteins are specific and have a particular meaning. A PPI network is structured as a weighted undirected network which models proteins as its nodes and models the interactions and interaction strengths between proteins as its edges and weights. There are different online sources for PPI, and in this article, we use PPI from STRING at https://string-db.org/, which provides relatively denser functional linkage networks.

Paired Expression and Chromatin Accessibility (PECA) provides a useful framework for modelling gene regulation from bio-sample matched and cell-type matched RNA-seq and DNase-seq data (Duren *et al.*, 2019). The Gene Regulation Network (GRN) inferred by PECA consists of four types of nodes: cis-Regulatory element (RE), chromatin regulator (CR), Transcription Factor (TF) and Target Gene (TG), and three types of edges (CR recruiting RE, TF binding to RE and RE regulating TG). The GRN provides a detailed view of how *trans*- and *cis*-regulatory elements work together to affect gene expression in a context-specific manner. The GRN of mouse and human used in this article can be downloaded from http://web.stanford.edu/~zduren/PECA/.

### 2.6 Evaluating performance of embedding generated by gene function prediction

We evaluate the performance of heterogeneous network embedding on gene function annotation using GOA for genes as gold standard positives. We split the existing GOA database into two parts. One is used to embed the network and the other to test the accuracy of the embedding. There are 607 460 annotations of human in AmiGO (http://amigo.geneontology.org/amigo/search/annotation) while half of them are repeated. We filter the redundant annotations and randomly divide them into a train set (80%) and a test set (20%).

### 2.7 RE-GOA-based functional enrichment analysis of genomic regions

Based on the RE-GOA generated, we develop a tool for functional enrichment analysis of a set of genomic regions. GREAT (McLean *et al.*, 2010), a widely used method, analyzes functional significance of the *cis*-regulatory region set by explicitly modelling the vertebrate genome regulation landscape and using many rich sources of information. Although defines a ‘regulatory domain’ for each gene to associate genomic regions and improves functional interpretation compared with previous methods to a certain degree, GREAT ignores distal and many to many regulations among REs and genes.

Based on RE-GOA, for a given set of genomics regions, we only retain chromatin regions whose distance from the nearest RE is smaller than 1 kb, and associate these regions with GO term set by the nearest RE and its annotations. Similarly to GREAT, we compute ontology term enrichments using a binomial test that explicitly accounts for variability in RE by measuring the total fraction of the RE annotated for any given ontology term as follows.

$$P(t)=\underset{\mathrm{binom}}{\mathrm{Pr}}\left(k\geq n|N,\;p(t)\right)$$

where t is the term for test, N is the number of peaks remaining, k=|{g|t∈GOA(NRE(g))}|, NRE(g) denotes the nearest RE of peak g, and p(t) denotes the ratio of REs annotated by term *t* to all the REs having annotations in RE-GOA.

We adjust the calculated P value using the *BH correction* and sort terms according to the adjusted P value (FDR). Terms with smaller FDR are more enriched in the input region set.

### 2.8 Annotating TF via its binding regions from ChIP-seq functional enrichment analysis

Chromatin Immuno-Precipitation followed by high-throughput sequencing (ChIP-seq) is a broadly used technique for identifying TF binding sites genome wide (Park, 2009). To quantitatively evaluate the performance of RE-GOA-based functional annotation of TFs, we compare functional enrichment results with Cistrome-GO as it shows high performance over other methods (Li *et al.*, 2019). We use BP terms annotating the evaluated TFs in AmiGO as the gold standard. Given a functional enrichment analysis from Cistrome-GO or RE-GOA, we define a threshold for the methods to be compared and the terms whose *FDR* is smaller than the threshold are selected. Terms with FDR corrected *P*-values are selected for evaluation, meaning:

$$T_{F}=\{t|p_{F}(t)<\theta \}$$

in which θ is the chosen threshold, $p_{F}(t)$ denotes the FDR-corrected P value of term t given the ChIP-seq peaks of F.

Same as Cistrome-GO, for each of these top enriched terms, its semantic similarity with each of the standard BP terms is calculated using GOGO (Zhao *et al.*, 2018), and the maximum semantic similarity (MSS) is used to measure the similarity between the enriched BP term and the TF BP standard terms. Precision, recall and $f_{1}\;\mathrm{value}$ are also considered as important measures. The measures used for evaluation include,

$$\mathrm{AvgMSS}=\Sigma_{t_{a}\in T_{\mathrm{predict}}(F)}\underset{t_{b}\in T_{\mathrm{standard}}(F)}{\max}\mathrm{sim}(t_{a},t_{b})$$

 

$$\mathrm{pre}=\frac{\left|T_{\mathrm{predict}}(F)\cap T_{\mathrm{standard}}(F)\right|}{\left|T_{\mathrm{predict}}(F)\right|}$$

 

$$\mathrm{rec}=\frac{\left|T_{\mathrm{predict}}(F)\cap T_{\mathrm{standard}}(F)\right|}{\left|T_{\mathrm{standard}}(F)\right|}$$

 

$$f_{1}=\frac{2*\mathrm{pre}*\mathrm{rec}}{\mathrm{pre}+\mathrm{rec}}$$

where $T_{\mathrm{predict}}(F)=\cup_{t\in T_{F}}anc\left(t\right)\cup T_{F}$ denotes the predicted result, $T_{\mathrm{standard}}(F)=\{t|t\in \mathrm{GOA}(F)\}$ denotes the standard result, and anc(t) is the set of term t's ancestors in GO. We compare our results with Cistrome-GO following its codes on https://bitbucket.org/liulab/cistrome-go/src/master/.

### 2.9 Functional enrichment analysis of differential genomic regions from ATAC-seq data

Assay for Transposase-Accessible Chromatin with high-throughput sequencing (ATAC-seq) is a powerful and widely used technique to measure genome-wide chromatin accessibility (Buenrostro *et al.*, 2015). Differential ATAC-seq analysis aims at identifying the difference in chromatin accessibility between two different conditions.

The tool which associates peaks with nearest annotated regulatory elements can be developed for functional enrichment analysis of the differential peaks. After significantly differential accessible peaks are obtained from two ATAC-seq data, RE-GOA-based functional enrichment analysis procedure is carried out to analyze difference in chromatin accessibility in two conditions.

### 2.10 Revealing genetic correlation among traits by function similarity

We use enriched terms by RE-GOA of associated SNPs from two traits to reveal their genetic correlation. For a given set of SNPs associated with a certain trait, we can conduct functional enrichment analysis based on RE-GOA, with which the enriched terms can help us to better understand the trait. After obtaining the enriched terms of a trait, we focus on associations between traits. In this article, we calculate the Jaccard similarity between two enriched term sets of two traits as their correlation, i.e.:

$$\mathrm{Sim}(T_{1},\;T_{2})=\frac{\left|{|S}_{1}\cap \;S_{2}|\right|}{\left|{|S}_{1}\cup \;S_{2}\right||}$$

where $T_{1}$ and $T_{2}$ are two traits, and $S_{1}$ and $S_{2}$ are the sets of enriched terms of the SNPs associated with $T_{1}$ and $T_{2}$, respectively.

We conduct genetic correlation analysis on 206 GWAS summary statistics which are selected from 4176 phenotypes and 11 372 GWAS summary statistics from UK-Biobank (https://www.ukbiobank.ac.uk/) based on the following conditions.

1. Sample size is larger than 50 000, and the size of samples for both binary and categorical phenotypes is larger than 10 000.
2. More than 500 significant SNPs that pass $5\times 10^{-8}$ threshold.
3. Sex-specific and ‘raw’ type GWAS are excluded.
4. Duplicated phenotypes and phenotypes associated with ‘job’, ‘parent’ or ‘sibling’ are removed.

We compare our results with another genetic correlation detection method, LDSC and the genetic correlation calculated by LDSC is downloaded from https://ukbb-rg.hail.is/.

## 3 Results

### 3.1 RE-GOA can effectively integrate information from different networks

Three biological networks, GRN, PPI and GOA, are integrated into a heterogeneous network with four types of nodes and seven types of edges. Statistics about the heterogeneous networks are listed in Supplementary Table S1. To capture the information in the heterogeneous network, multiple meta-paths are defined. Each meta-path is an ordered sequence of node types that connects two nodes with a relation. To demonstrate that RE-GOA can effectively integrate information by embedding the heterogeneous network, we test whether the embedding vectors for genes are informative about gene functions. We extract genes’ annotations in GOA database as gold standard which allows us to benchmark the performance by accuracy.

We first quantitatively evaluate the relative contributions of the different data components in our heterogeneous network. We compare the performance of gene function prediction based on different combinations of input data (described in Section 2). One is the model that embeds a heterogeneous network which integrates GOA, PPI and GRN. The other two only integrate GOA and PPI, and GOA and GRN, respectively. The area under the receiver operator characteristics (AUROC) is calculated. The larger the AUROC is, the better the model performs. The results (Fig. 2b–d) show that both PPI and GRN are predictive and all of the three subnets (GOA, PPI, GRN) are necessary and contribute to the model. The model combining GOA and PPI performs better than the one combining GOA and GRN in BP but not in CC. RE-GOA performs the best by integrating all of the three data sources. This demonstrates that our integrative strategy is effective and different components of the heterogeneous network are complementary to each other.

We next demonstrate that the choice of meta-paths can indeed capture the local and global structure in the network and assess the contributions from different meta-paths. The meta-paths can be divided into three subsets using different relationships within PPI, GO or GRN. The separated experiments have been conducted and the results (Fig. 2b–d) show that both PPI and GRN are predictive and all of the three subnets (GO, PPI, GRN) are necessary and contribute to the model. Four meta-paths are defined to capture the regulatory relations in GRN. As discussed above, RT links REs with terms directly according to the annotation of genes which are regulated by the REs; the meta-paths *FRGRF* and *RGGR* both capture the regulatory relationship between *cis*-regulatory elements and target genes; and the meta-path *FRF* captures the relationship of TF binding to RE. We compare the models with different meta-path combinations to demonstrate their effectiveness based on gene function prediction (Table 1). The meta-path RT is shown to be crucial since the accuracy reduces a lot without it in all three GO categories (Fig. 2b–d, Table 1). FRF improves accuracy in MF and CC more than in BP when compared with the compound meta-paths. It is worth noting that using solely the direct path RT or one of the indirect paths (FRF, RGGR and GRFRG) decreases the accuracy of gene function prediction in BP, while their combination promotes accuracy. Based on these observations, all of the meta-paths are integrated, resulting in overall the highest accuracy.

**Table 1.** Comparing different meta-path combinations in gene function prediction

Meta-paths	Combination of meta-paths					RE-GOA
RT	×	×	√	√	√	√
FRF	×	√	×	×	√	√
GRFRG + RGGR	×	√	×	√	×	√
AUROC						
BP	0.889	0.829	0.86	0.907	0.872	0.905
MF	0.905	0.872	0.949	0.95	0.956	0.952
CC	0.747	0.766	0.925	0.91	0.922	0.913

Finally, we demonstrate that RE-GOA can capture the distal regulation in the heterogeneous network. The GRN modeled by PECA includes both proximal and distal REs with distance to the TSS of its target gene up to 500 kb. According to the distance of RE to TSS of genes, we divide the regulations among REs and genes into proximal regulation (within 5 kb) and distal regulation. We then carry out gene function prediction with either proximal or distal regulations in GRN. Figure 2e shows that both of them are critical to the model. Although the proximal regulations only account for a small part of the whole GRN (0.2%), the absence of them results in about 0.05 decline of the AUROC. The model with proximal regulations even performs better than the model with distal regulation relations only. Figure 2f indicates that both of the annotations inferred by distal and proximal regulations provide correct annotations. Distal part provides more than proximal part in MF, whereas proximal part contributes more in BP and CC.

### 3.2 RE-GOA provides a genome wide resource for RE’s ontology annotation in human and mouse

After making sure that network embedding in heterogeneous network can correctly capture information, we apply RE-GOA to generating a resource for RE annotation in human (hg19) and mouse (mm9). Supplementary Table S4 lists some statistics of the resource. Based on this RE-GOA generated resource, we develop a procedure for functional enrichment analysis of genomic regions. For a given set of genomic regions, we only retain chromatin regions whose distance from the nearest RE is within ±1 kb, and associate these regions with term set by the nearest RE and its annotation. Similarly to GREAT (McLean *et al.*, 2010), we compute ontology term enrichments using a binomial test that explicitly accounts for variability in RE by measuring the total ratio of the REs annotated by any given ontology term to all the REs annotated in RE-GOA. Supplementary Figure S2 shows a brief workflow of the functional enrichment analysis based on our RE-GOA, and the details can be found in Section 2.

In order to demonstrate the accuracy and performance of the RE-GOA and RE-GOA-based functional enrichment analysis of genomic regions, we next apply it to three different biological data analysis tasks, including annotating TFs via their binding regions from ChIP-seq data in different tissues, analyzing differential accessible peaks of ATAC-seq data, and estimating GWAS summary statistics-based phenotype similarities.

### 3.3 RE-GOA outperforms Cistrome-GO in annotating TF binding sites from ChIP-seq data

**Fig. 3.**
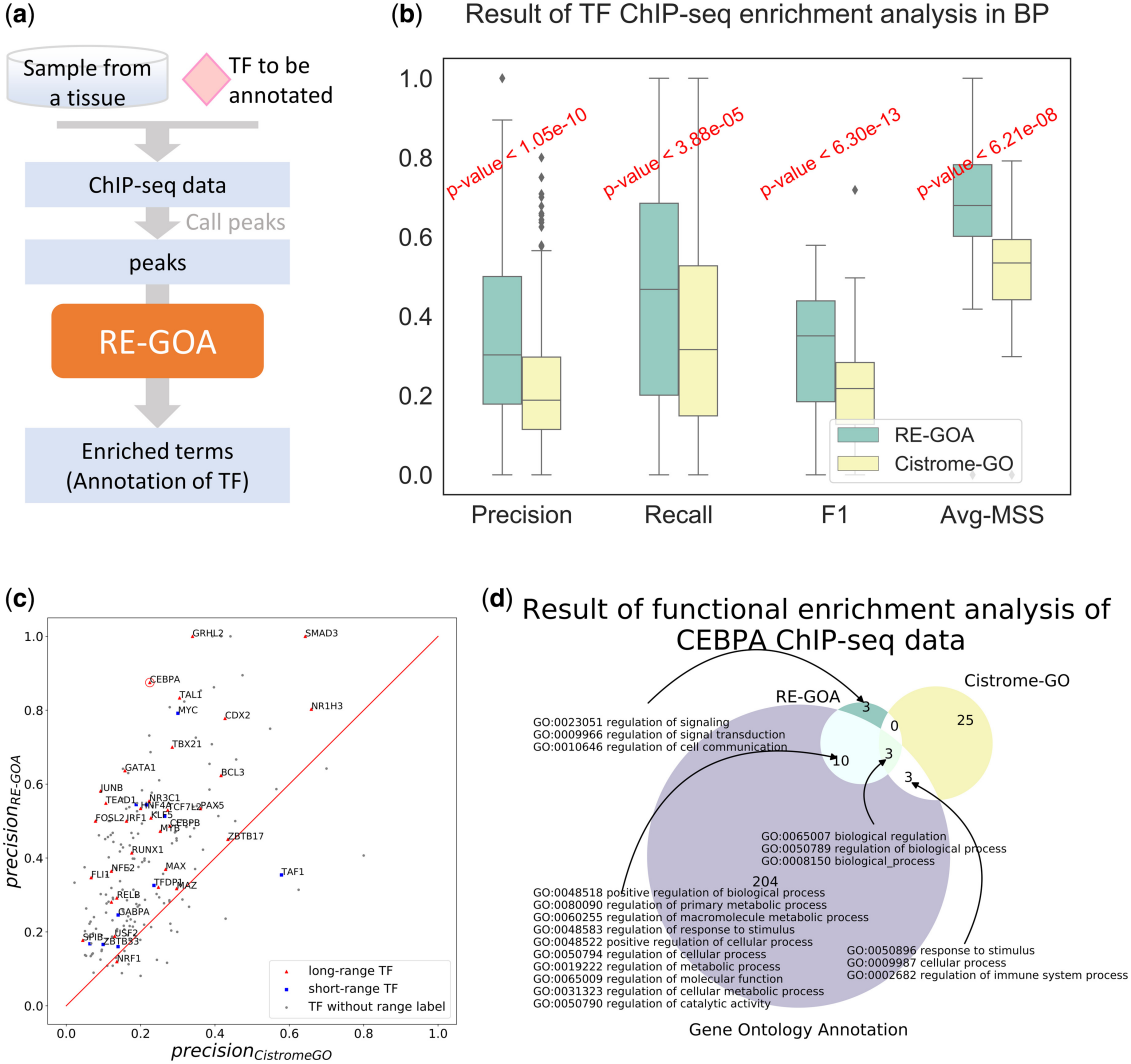
RE-GOA annotates TFs via their binding regions from ChIP-seq data. (**a**) Workflow of RE-GOA-based annotating TF via its binding regions from ChIP-seq data. Taking TF ChIP-seq data in a certain tissue as input, RE-GOA annotates TFs via their binding regions’ enriched function. (**b**) The boxplot of precision, recall, f1-value, and Average Maximum Semantic Similarity (Avg-MSS) of RE-GOA and Cistrome-GO in 247 TFs’ ChIP-seq data. RE-GOA outperforms Cistrome-GO in TFs’ functional enrichment analysis. (**c**) Scatter plot of precision of RE-GOA and Cistrome-GO for 247 TFs, including both long-range TFs and short-range TFs. (**d**) Comparison of CEBPA’s ChIP-seq enrichment analysis in GE-GOA and Cistrome-GO with its annotation in GO database as gold standard. Given CEBPA ChIP-seq data from Pleura, we take out the top 10 enriched terms of RE-GOA and Cistrome-GO. The 10 terms and all of their ancestors are compared with the gold standard: annotation in AmiGO

The ChIP-seq data describe the TF binding sites in a genome wide way (Park, 2009). Our functional enrichment analysis tool based on RE-GOA can be used for ChIP-seq data analysis, and Figure 3a shows a workflow for RE-GOA annotating TFs via their binding regions from ChIP-seq data. The genomic regions obtained from ChIP-seq data are taken as input for this procedure, and the enriched terms are output for annotating TFs’ function.

To quantitatively evaluate the performance of RE-GOA, we extract ChIP-seq datasets of 247 human TFs available in the Cistrome data base (http://cistrome.org/db/#/) which were collected by Li *et al.* (Li *et al.*, 2019) and the standard GO annotation available in AmiGO (Carbon *et al.*, 2009). For each TF, the largest number of peaks is used for comparison. For each TF ChIP-seq dataset, we use the top 15 000 peaks **(**ranked by -log⁡Pvalue, if the peak number is larger than 15k**)** or all peaks (if peak number is less than 15k). Supplementary Figure S3a shows a distribution of peaks’ distances to the nearest RE. Averagely over 60% of the peaks are in 1 kb distance from REs, which means that most of the peaks have been annotated by an RE in our method. The ChIP-seq peak file is input for both RE-GOA and Cistrome-GO (Li *et al.*, 2019), and we calculate AvgMSS, precision, recall and $f_{1}\;\mathrm{value}$ as evaluation indexes (described in Section 2). In order to balance precision and recall, we define different thresholds for the two methods: $5\times 10^{-8}$ for RE-GOA and $5\times 10^{-2}$ for Cistrome-GO. The median performance of RE-GOA is better than that of Cistrome-GO in all four accuracy indicators (BP: Fig. 3b, MF: Supplementary Fig. S2b, CC: Supplementary Fig. S2c). RE-GOA improves the four indicators up to 30% on average in comparison with Cistrome-GO.

Chen *et al.* (2020) divided TFs into two types, long-range and short-range TFs, and offered a resource of regulatory ranges of TFs. Figure 3c shows the stability of RE-GOA on different types of TFs. RE-GOA provides higher precision than Cistrome-GO in 31 out of 32 long-range TFs and 9 of 10 short-range TFs, which indicates that RE-GOA performs well in both long-range and short-range TFs.

Cellular identity is primarily regulated by TFs to recognize and bind to context specific sequences in the genome for regulating gene expression. D’Alessio *et al.* (2015) identified candidate core TFs across different cell types. We mark TFs which are specific in their ChIP-seq sample tissues. As shown in Supplement Figure S4a, RE-GOA outperforms Cistrome-GO in all of the tissue specific TFs. Supplementary Figure S4b–h shows tissues with more than 10 samples and non-zero tissue specific TFs.

*CEBPA* is known as the main epithelial ‘gatekeeper’. Its expression is necessary to prevent unnecessary mesenchymal transition and supports the important role of epithelial–mesenchymal transition in mediating breast cancer metastasis (Lourenço *et al.*, 2020). We extract *CEBPA* ChIP-seq data of Pleura from Trompouki *et al.* (2011) and annotate its function by RE-GOA. Figure 3d shows a comparison of RE-GOA and Cistrome-GO for *CEBPA* ChIP-seq top 10 enriched terms. RE-GOA provides better results for *CEBPA* ChIP-seq data analysis than Cistrome-GO. Thirteen out of the all 220 standard terms are enriched by RE-GOA whereas 10 of them are not found by Cistrome-GO. Moreover, RE-GOA provides more precise and specific results than Cistrome-GO. Besides the terms which annotate *CEBPA* in AmiGO, terms about signaling and cell communication such as *GO: 0023051 regulation of signaling* and *GO: regulation of cell communication* are enriched in the RE-GOA results, and are closely associated with samples from Trompouki *et al.* (2011). This demonstrates that RE-GOA can capture not only TF’s function but also cellular context features from samples.

### 3.4 RE-GOA presents more reasonable differential ATAC-seq analysis results than GREAT

**Fig. 4.**
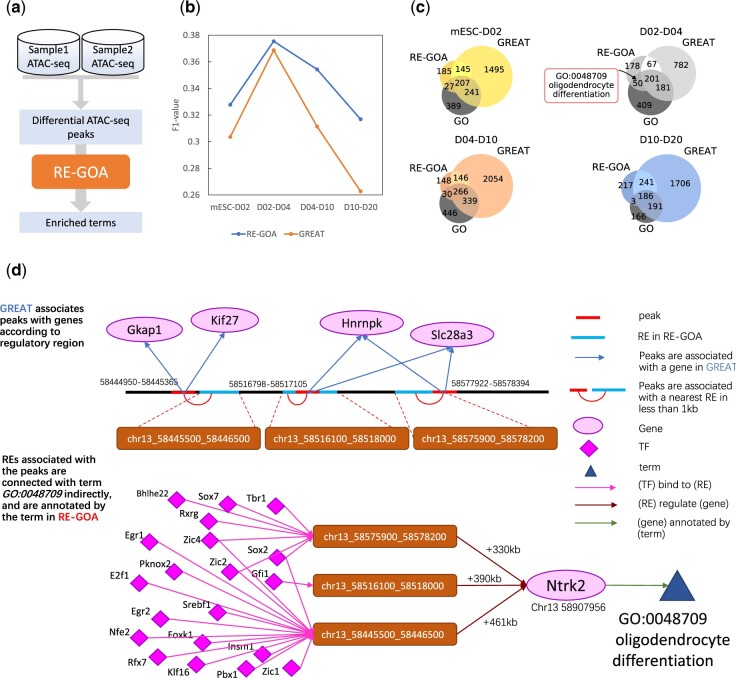
RE-GOA performs functional enrichment analysis of differential genomic regions from ATAC-seq data. (**a**) Workflow of RE-GOA-based functional enrichment analysis of differential genomic regions from ATAC-seq data. Given ATAC-seq data in two different conditions, we first extract the differential peaks by comparing the two ATAC-seq data, and then conduct RE-GOA-based functional enrichment analysis on these differential peaks. (**b**, **c**) Comparison of RE-GOA and GREAT in BP. Taking GO enrichment analysis of differential expressed genes between two time points as gold standard, RE-GOA provides higher f1-value over GREAT. (**d**) GO:0048709 is enriched in REGOA’s result but missed by GREAT. RE-GOA could associate peaks with nearest annotated Res which regulate gene Nkrt2 in GRN, and are annotated by term GO:0048709 in RE-GOA. While GREAT associates peaks with nearest genes, which do not have annotation GO:0048709 in AmiGO. To summary, GO:0048709 is enriched in RE-GOA’s results and absent in GREAT’s

ATAC-seq is used widely for studying genome-wide chromatin accessibility (Buenrostro *et al.*, 2015), and differential ATAC-seq analysis are designed aiming at analyzing chromatin accessibility of samples from different conditions. RE-GOA can be used for functional enrichment analysis of the differential peaks. A workflow for differential ATAC-seq analysis is shown in Figure 4a and the details have been discussed in Section 2.

We perform this procedure on ATAC-seq data of retinoic acid-induced mESC cells at days 0, 2, 4, 10 and 20 (mESC, D2, D4, D10 and D20) (Duren *et al.*, 2020). We select peaks whose openness rate between two time points is greater than a selected threshold **(**which means $\frac{O_{t_{1}}}{O_{t_{2}}}>\phi$ or $\frac{O_{t_{2}}}{O_{t_{1}}}>\phi$, where $O_{t_{1}}$ and $O_{t_{2}}$ represent openness at $t_{1}$ and $t_{2}$, respectively, and here, we selected ϕ=2), and at least at one time point openness is larger than 2 **(**which means $O_{t_{1}}>2$ or $O_{t_{2}}>2$). The number of differential peaks is listed in Supplementary Figure S5a. There are over 20 000 differential peaks in day 0 versus day 2, day 4 versus day 10 and day 10 versus day 20, and about 7000 differential peaks for day 2 versus day 4.

**Fig. 5.**
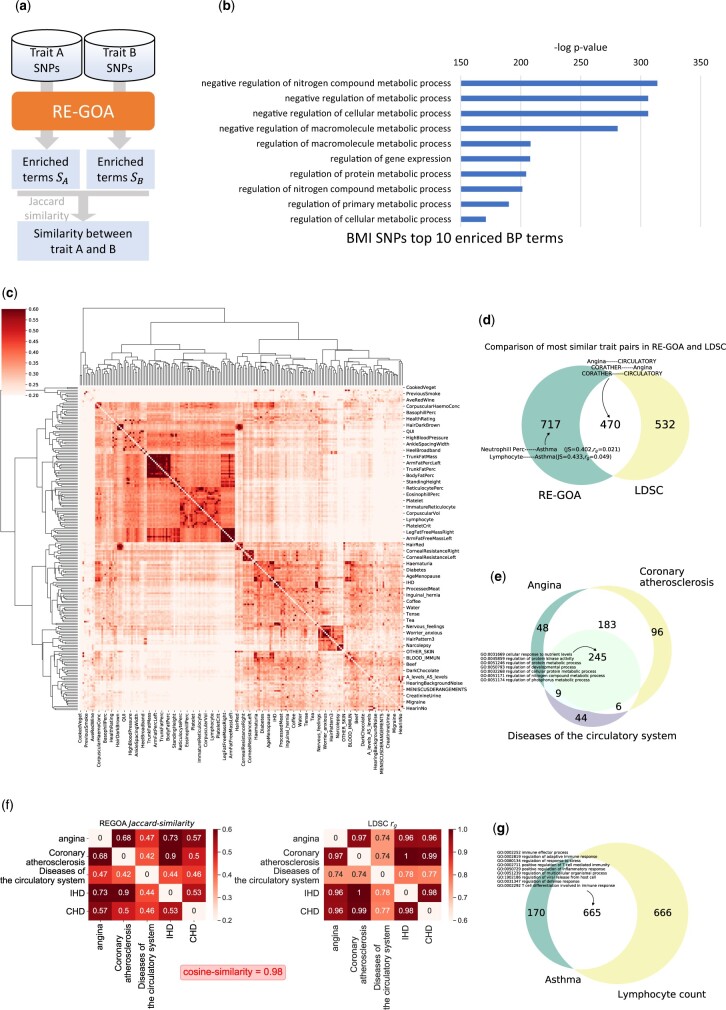
RE-GOA reveals genetic correlation among phenotypes from their GWAS summary statistics data. (**a**) Workflow of RE-GOA-based genetic correlation inference from GWAS data. Given two traits’ GWAS summary statistics, we first use RE-GOA-based functional enrichment analysis to obtain a set of GO terms for each trait. Then we calculate the Jaccard similarity between two GO term sets as the similarity of the two traits. (**b**) BMI’s top 10 enriched BP terms. Given SNPs of BMI, RE-GOA reveals metabolic process related terms. (**c**) Heatmap of similarity among traits from UKBiobank. A clustered heatmap for RE-GOA-based GWAS trait similarity is shown. (**d**) Comparison of the most similar trait pairs in RE-GOA and LDSC. (**e**) Similar trait pairs are found both by RE-GOA and LDSC sharing many common enriched terms. Terms about metabolic process are enriched in RE-GOA-based enrichment analysis for heart and circulatory system. (**f**) Heatmap for similarity calculated by RE-GOA and LDSC among traits shows have high similarity. (**g**) Traits with low similarity calculated by LDSC have high similarity in RE-GOA. Asthma is known as a type of autoimmune disease. Immunity related GO terms are enriched. In RE-GOA, asthma has a high similarity with Lymphocyte count because they are both associated with immunity

After obtaining the differential peak sets, we perform enrichment analysis using RE-GOA and compare with GREAT. Supplementary Figure S5b shows that most of the peaks are located in 1 kb near the REs. In order to evaluate the performance of RE-GOA quantitatively, differentially expressed genes (DEGs) are calculated from matched RNA-seq data by comparing adjacent time points and the Gene Set Enrichment Analysis (GSEA) results for those DEGs are used as gold standard. We pick out the terms with P value<0.05 in the enrichment analysis by GREAT and RE-GOA. We then compare their results after obtaining all of the term's ancestors as shown in Figure 4b and c and Supplementary Figure S5c. RE-GOA outperforms GREAT with higher precisions and f1 value at all four time points.

Some important GO terms are identified. For example, *GO:0048709 oligodendrocyte differentiation* is enriched in GSEA result, indicating that a crucial process takes place at day 2 to day 4 of RA-induced mESC differentiation. This term is also enriched in the results of D2–D4 peaks provided by RE-GOA. As shown in Figure 4d, RE-GOA associates three peaks with REs which are annotated by *GO:0048709*. These REs regulate gene *Ntrk2* in GRN, which is located up to 461 kb downstream of the REs, whereas this distal regulation is hard to be found by GREAT.

### 3.5 RE-GOA reveals genetic correlation among phenotypes from their GWAS summary statistics data

A large number of resources and methods have been developed for Genome-Wide Association Study (GWAS) (Visscher *et al.*, 2017) for complex traits, which are highly polygenetic and pleiotropic. While the limited capacity in performing large-scale evaluation of functional impact thwarts the understanding of biological mechanisms (Albert *et al.*, 2015), RE-GOA could be used for analyzing GWAS summary statistics. A framework for RE-GOA-based trait similarity calculation is shown in Figure 5a, and the details are described in Section 2. Given a set of SNPs, RE-GOA could find enriched GO terms associated with the corresponding trait, which may reveal the mechanism between genes and traits. We define similarity between two traits as the Jaccard similarity of their enriched term sets.

We conduct experiment on 206 traits from UK-Biobank (https://www.nealelab.is/uk-biobank) (details for selecting statistics are presented in Section 2). Supplementary Figure S6b shows that RE-GOA could capture features of about 30% of the SNPs. Figure 5b lists the top 10 enriched terms of trait *BMI* calculated by RE-GOA, and one can see that most of them are related to metabolic process. This demonstrates that RE-GOA can provide reasonable interpretations for the traits based on their SNPs. As comparison, only 1 of 4 terms enriched in the GREAT results is related with metabolic process (Supplementary Fig. S6a). A heatmap is shown in Figure 5c for genetic correlation estimation for all 21 321 pairwise combinations of the 206 traits. Supplementary Figure S6c shows that trait similarities concentrate on values from 0.2 to 0.4. We also calculate traits’ genetic correlation $r_{g}$ using LDSC (Bulik-Sullivan *et al.*, 2015). Supplementary Figure S6d shows that the trait similarities using LDSC concentrate on the values around 0. Based on these observations, we compare trait pairs whose RE-GOA similarity is larger than 0.4 with those whose $r_{g}$ calculated by LDSC is larger than 0.4. As Figure 5d shows, common pairs and different pairs between RE-GOA and LDSC both account for a large part. Traits related to diseases of heart and circulatory system including *Angina*, *Coronary atherosclerosis*, *Diseases of the circulatory system*, *IHD (Ischemic Heart Disease)* and *CHD (Coronary Heart Disease)* have high similarity in both RE-GOA and LDSC (Fig. 5e and f). Terms about metabolic activities such as *GO:0051246 regulation of protein metabolic process* are enriched in RE-GOA-based enrichment analysis of the three traits, *Angina*, *Coronary atherosclerosis* and *Diseases of the circulatory system*. The similarities **(**Jaccard Similarity and $r_{g}$) between traits calculated by RE-GOA and LDSC respectively have a cosine similarity up to 0.98, which indicates that both methods could capture the genetic correlation among the traits. *Coronary atherosclerosis* and *IHD (Ischemic Heart Disease)* have high similarity calculated by both RE-GOA (Jaccard similarity = 0.9) and LDSC ($r_{g}$=1**)**, and the links between them have been studied and confirmed in the past years (Ahmadi *et al.*, 2016; Marzilli *et al.*, 2012). *Asthma* and *Lymphocyte count* are not identified as genetically correlated with $r_{g}=0.049$ calculated by LDSC, while terms about immune system are enriched in both of their SNPs enrichment analysis result (Fig. 5g). Asthma is known as a self-immune disease, and the correlation between asthma disease with lymphocyte type and neutrophil to lymphocyte ratio is well studied (Gungen *et al.*, 2017; Moldaver *et al.*, 2017; Schuyler *et al.*, 1981). In summary, RE-GOA provides reasonable results in revealing genetic correlation among phenotypes from their GWAS summary statistics data.

## 4 Discussion and conclusions

In this article, we propose a framework (RE-GOA) for assigning gene ontology annotation to cis-regulatory elements via heterogeneous network embedding. This expands the traditional ‘genes’ eye view. Based on the annotation that RE-GOA generates, we perform functional enrichment analysis of specific gene region set, and use it in three different biology applications including annotating TF binding sites from ChIP-seq data, analyzing differentially accessible peaks from ATAC-seq data, and revealing genetic correlation of phenotypes from GWAS data. Our major contribution consists of a heterogeneous network embedding method by integrating context specific regulatory networks with PPI and gene ontology hierarchical structures. When utilized in biological applications, RE-GOA outperforms the existing methods in studying ChIP-seq, ATAC-seq and GWAS data. Moreover, we systematically generate a resource for RE ontology annotation in human and mouse.

The method for functional enrichment analysis based on RE-GOA has unique features compared with other peak analysis tools such as GREAT. One of the most prominent features is to use the context specific distal regulations of REs rather than associate peaks with ‘the nearest gene’. This is significant since REs can stimulate gene activity via long genomic distances. Besides that, RE-GOA integrates GRN, PPI and GOA into a heterogeneous network and holds the promise to provide more accurate annotation. Importantly, taking advantage of embedding techniques from NLP makes it possible to embed heterogeneous network into vector space, which allows latent information mining. And we have shown that combining the diverse networks can effectively improve the performance.

Gene Ontology categorizes and defines different relations among terms and the commonly used relationships include: ‘is_a’, ‘part_of’, ‘has_part’ and ‘regulates’ (includes ‘negatively regulates’ and ‘positively regulates*’*). We use only ‘is_a’ relationship in our study and neglect others such as ‘part_of’ and ‘regulates’ considering their limited coverage in Gene Ontology. All terms (except the root terms representing categories themselves) have an ‘is_a*’* sub-class relationship to another term, and ‘is_a’ is involved in most reasoning rules for defining meta-paths. As shown in Supplementary Table S2, only a small part of the GO terms have ‘part_of’, ‘has_part’ and ‘regulates’ relations with others and a continuous meta-path can hardly be defined based on these relationships. Moreover, sometimes these relationships occur in cross-subontology connections between two different subontologies. For example, 1,068 ‘part_of’ relations link a MF term to a BP term; 844 ‘regulates’ relations claim that a BP term regulates a MF term. In our study, we construct heterogeneous network with the three parts of Gene Ontology separately, and the cross-subontology relations are neglected. We further conduct experiments using all of the relationships within the same sub-ontology. We extend the ‘is_a’ relationship in the defined meta-paths with all the relationships in GO, and validate the network in gene function prediction. The result show (Supplementary Table S3) that simply merging all of the relationships reduces the AUROC in MF and CC more than its improvement in BP.

Regulatory network construction is crucial by emphasizing RE’s function in long distance regulation and in specific cellular contexts. The gene regulatory network we used in our RE-GOA connects REs to their target genes according to their co-activity across samples with paired gene expression and chromatin accessibility data instead of assigning REs to their nearest genes. In the similar spirit, regulatory network can be constructed by other omics data and methods, including modelling networks via enhancer-promoter DNA sequences, Hi-Chip data, eQTL correlation and multi-genomic data integration. For example, DC3 provides a method for joint analysis of various bulk and single-cell data and constructs gene regulatory network from deconvoluted Hi-Chip data, with which chromatin contacts between active REs and target genes are measured (Zeng *et al.*, 2019); EP2vec uses NLP methods and predicts enhancer-promoter interactions from three-dimensional genomic interactions (Zeng *et al.*, 2018); a two-sample SMR + HEIDI framework uses eQTL data from GTEx and the eQTLGen project takes a deep insight into tissue-specific transcriptional mechanisms (Richardson *et al.*, 2020). All of these gene regulatory networks constructed by different methods are adaptable for our RE-GOA framework.

The network embedding is used widely in bioinformatics, and various embedding methods have been developed in the past years. However, most of them, such as graph convolution networks (GCNs) (Kipf *et al.*, 2017) or DeepWalk (Perozzi *et al.*, 2014), do not distinguish among node and edge types and turn the network into a homogeneous network. In our work, the heterogeneity enables the definition of meta-paths to encode domain knowledge, making the framework explainable and scalable for diverse data. However, due to the complexity of indirect regulations, the meta-paths maybe not be enough to capture all the potential relations among biological objects. In addition, the design of meta-paths for each type of heterogeneous networks can be biased by specific domain knowledge. For the next step, we will explore distinct ways to extract information from heterogeneous network without manually designing meta-paths. One possible direction is to define basic meta-relations to parameterize the weight matrices for calculating attention over each edge. This strategy can incorporate information from high-order neighbors of different types through message passing across layers, which can be regarded as ‘soft’ meta-paths (Hu *et al.*, 2020).

In our future work, RE-GOA can be extended to integrate more diverse biological data and to include gene regulatory networks constructed by other methods and pathway analysis, and more sophisticated algorithms can be developed for integrating data of high heterogeneity. In this study, only the types of nodes are considered for meta-path definition, while a strategy for properly using the different GO relationships remains to be proposed. In addition, due to the large number of nodes in the heterogeneous network, lengthy times have to be spent on training and annotating. Also, in the application for GWAS summary statistics, we can only identify the functional correlation relationships among traits, but fail to distinguish the causal relations. These are among the difficulties to be tackled in our future work.

## Supplementary Material

btac185_Supplementary_Data
