# Supplementary material for: Annotating regulatory elements by heterogeneous network embedding
Source: Bioinformatics. 2022 Mar 24;38(10):2899–911. doi: 10.1093/bioinformatics/btac185 (PMC9326849; doi:10.1093/bioinformatics/btac185)
Supplement: btac185_Supplementary_Data [file btac185_supplementary_data.pdf]

Table S1: Statistics about the constructed heterogeneous networks.

	GO part	Number of genes	Number of terms	Number of REs	Number of TFs
Human	MF	14,503	11,163	144,202	636
	BP	13,968	28,769		
	CC	14,443	4,185		
Mouse	MF	13,774	11,153	168,883	357
	BP	14,018	28,748		
	CC	14,189	4,184		

Table S2: Count of terms with different relations in GO

GO part	Term num	Num of terms with different realtions			
		part_of	*has_part*	*regulates*	*ends_during*
MF	11153	1056	194	101	0
BP	28748	4868	294	9575	9
CC	4184	1868	125	0	0

Table S3: AUROC when using different relatons in GO

AUROC	Only *is_a* relations used	All relations used
MF	0.952	0.951
BP	0.905	0.911
CC	0.913	0.905

Table S4: Statistics about RE-GOA

	GO part	term num	RE num	Avg num of terms annotating per RE	Avg num of REs annotated by per term
Human	MF	1,665	138,089	9.832	815.5
	BP	7,430	136,685	65.21	1,199.6
	CC	831	139,303	10.81	1,812.2
Mouse	MF	1,577	152,800	3.666	355.2
	BP	6,697	154,904	34.902	807.3
	CC	896	154,963	6.742	1,166.1

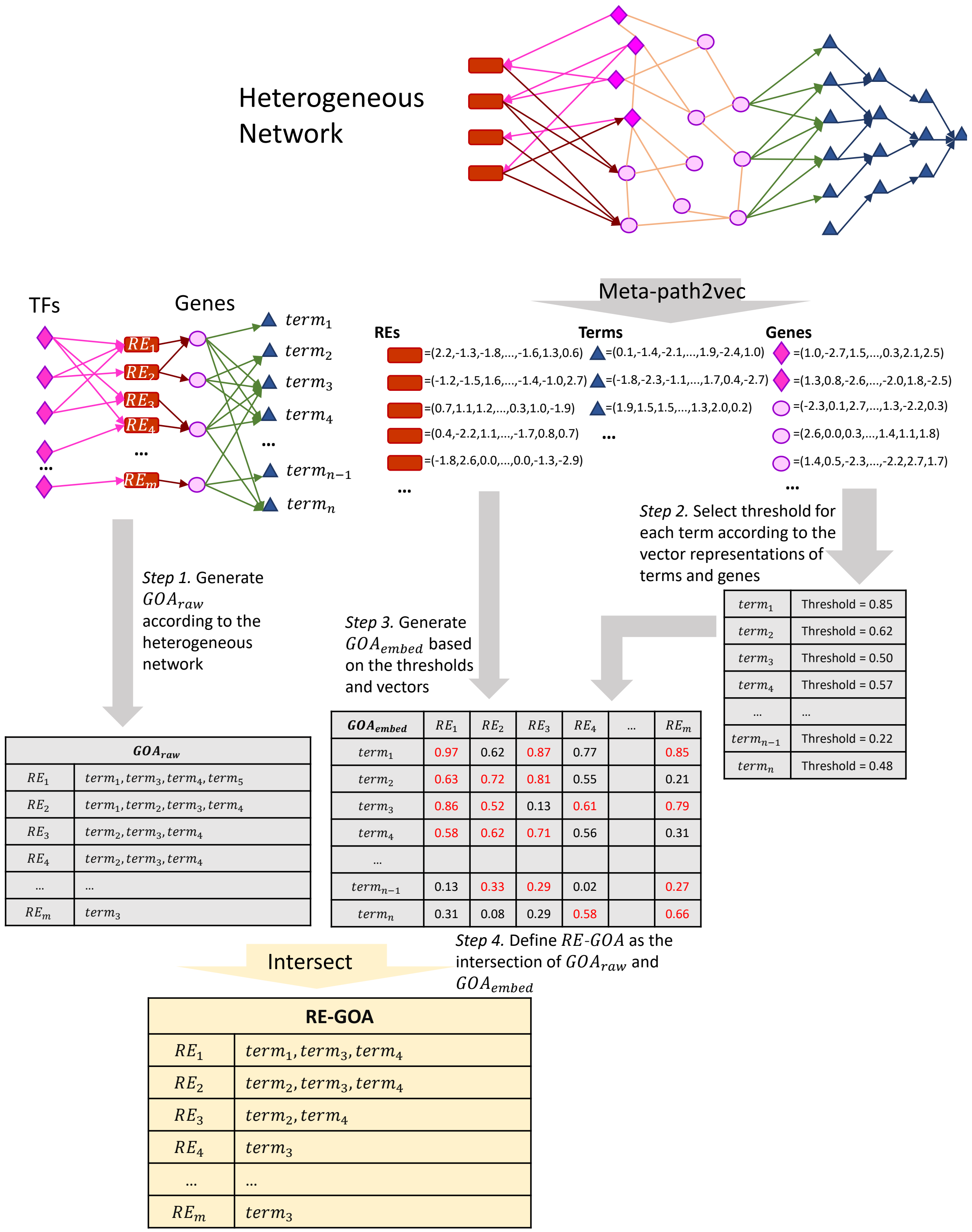

Fig. S1. Details for annotating REs based on the embedding generated form heterogeneous network. A proper threshold is selected for each term according to the genes it annotates and their embeddings. After calculating the threshold for each term, we annotate REs with terms according to the threshold and embedding. GOA_{embed} is selected out according to the thresholds and the vertor representations of terms and REs, and then filtered by GOA_{raw}.

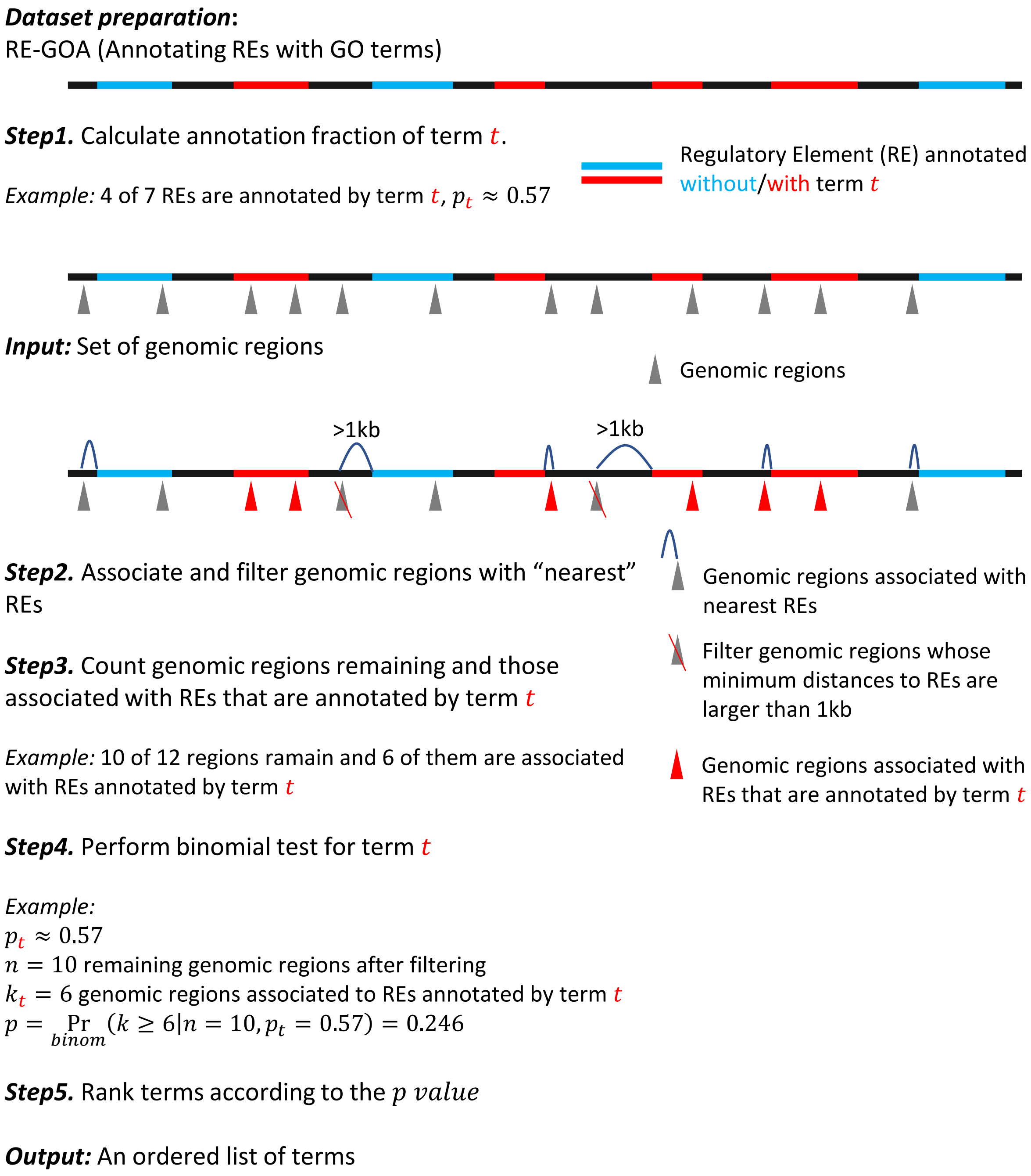

Fig. S2. Workflow of RE-GOA based genomic regions enrichment analysis

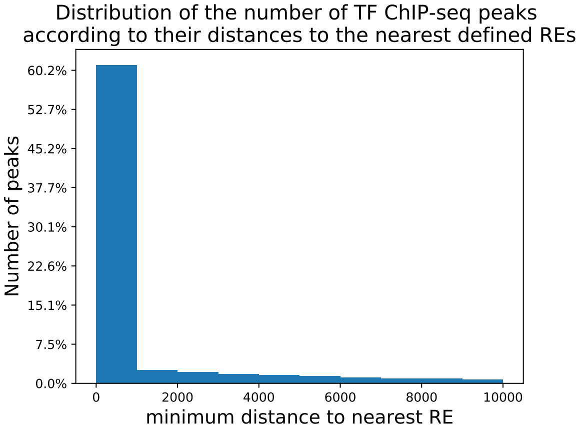

(a)

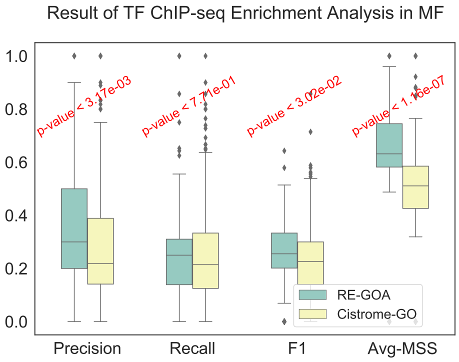

(b)

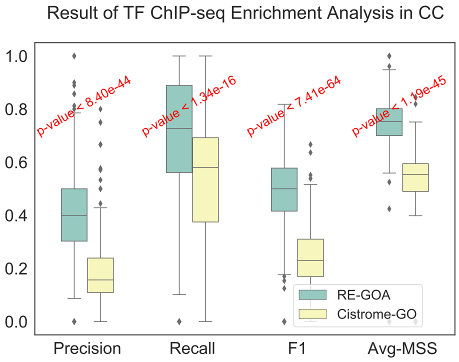

(c)

Fig. S3. RE-GOA outperforms existing methods in annotating TF via its binding regions form ChIP-seq data. (a) Distribution of TF ChIP-seq peaks. We use 247 TFs ChIP-seq data and for most of the peaks (60.2%), their distance to the nearest defined RE are less than 1kb. (b)(c) The boxplot of precision, recall, f1-value, and Average Maximum Semantic Similarity (Avg-MSS) of RE-GOA and Cistrome-GO in 247 TFs' ChIP-seq data in MF(b) and CC(c)

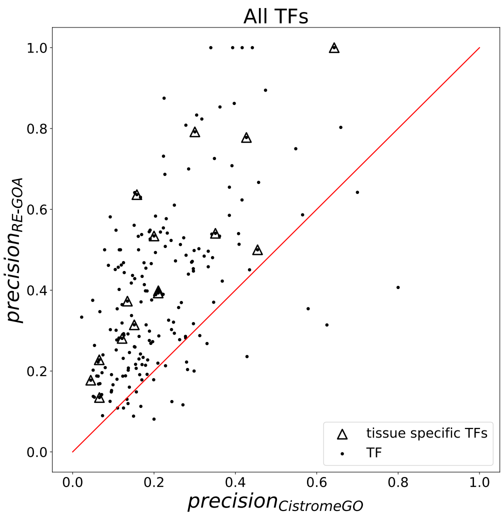
All TFs
precision_RE-GOA
precision_CistromeGO
tissue specific TFs
TF

(a)

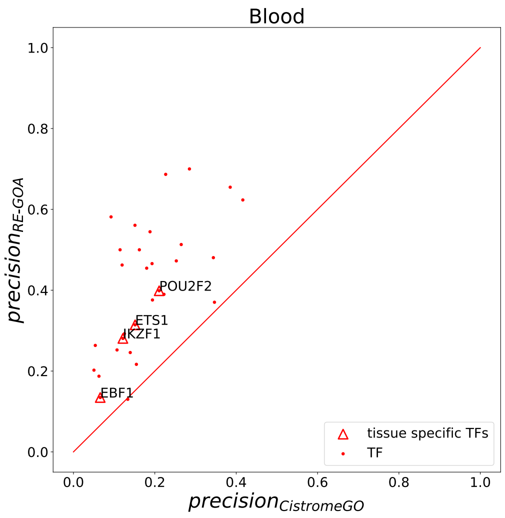
Blood
precision_RE-GOA
precision_CistromeGO
POU2F2
ETS1
IKZF1
EBF1
tissue specific TFs
TF

(b)

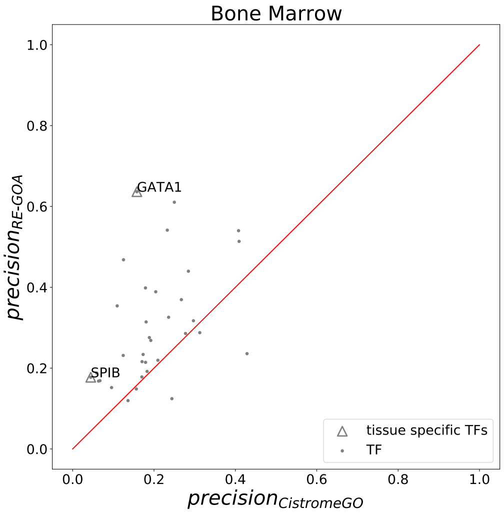
Bone Marrow
precision_RE-GOA
precision_CistromeGO
GATA1
SPIB
tissue specific TFs
TF

(c)

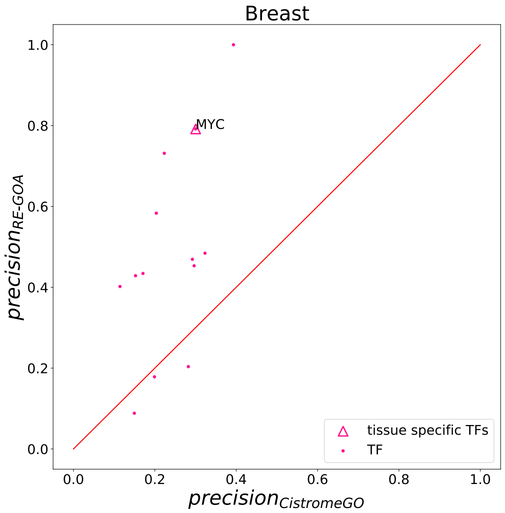
Breast
precision_RE-GOA
precision_CistromeGO
MYC
tissue specific TFs
TF

(d)

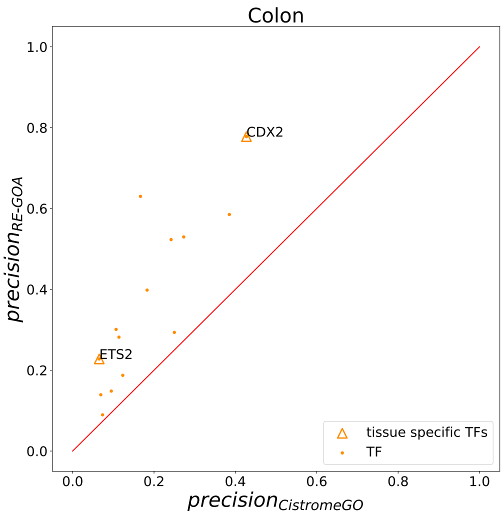
Colon
precision_RE-GOA
precision_CistromeGO
CDX2
ETS2
tissue specific TFs
TF

(e)

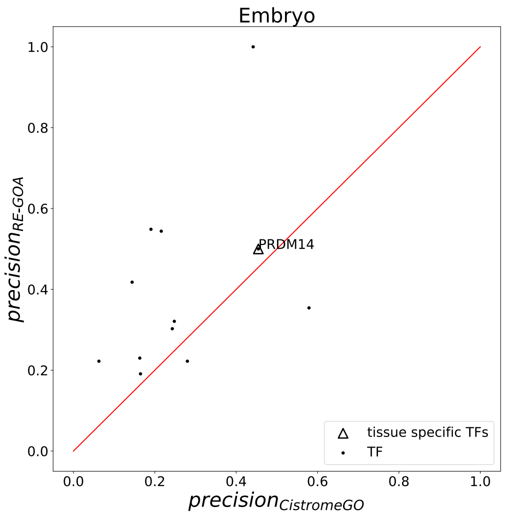
Embryo
precision_RE-GOA
precision_CistromeGO
PRDM14
tissue specific TFs
TF

(f)

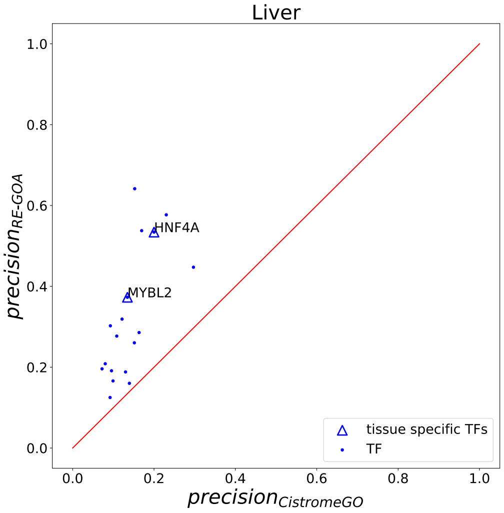

(g)

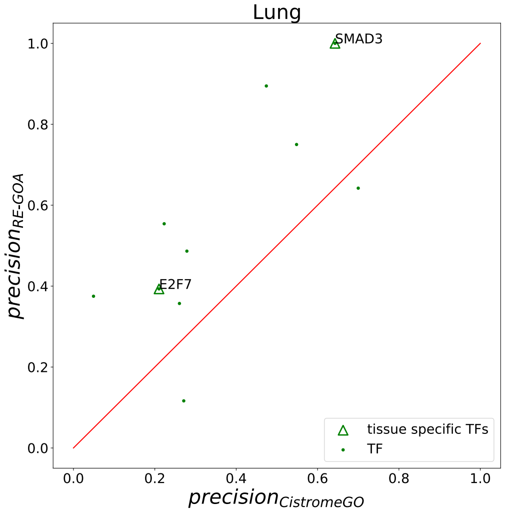

(h)

Fig. S4. RE-GOA outperforms Cistrome-GO in all of the tissue specific TFs.

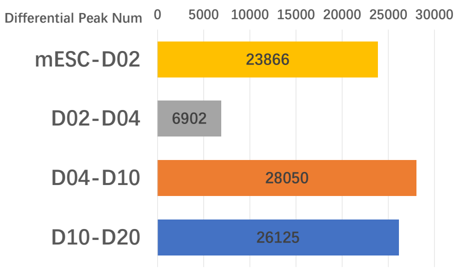

(a)

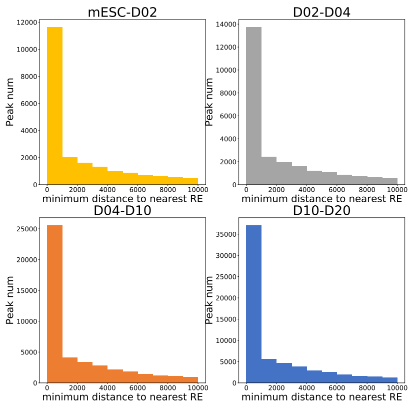

(b)

		precision	recall	f1-value	
mESC-D02	MF	0.288	0.218	0.248	RE-GOA
		0.099	0.474	0.164	GREAT
	BP	0.415	0.271	0.328	RE-GOA
		0.215	0.519	0.304	GREAT
	CC	0.355	0.212	0.265	RE-GOA
		0.132	0.442	0.203	GREAT
D02-D04	MF	0.351	0.547	0.427	RE-GOA
		0.264	0.383	0.313	GREAT
	BP	0.507	0.301	0.378	RE-GOA
		0.31	0.454	0.369	GREAT
	CC	0.786	0.122	0.211	RE-GOA
		0.471	0.182	0.263	GREAT
D04-D10	MF	0.396	0.315	0.351	RE-GOA
		0.154	0.601	0.246	GREAT
	BP	0.502	0.274	0.354	RE-GOA
		0.216	0.56	0.311	GREAT
	CC	0.568	0.127	0.208	RE-GOA
		0.256	0.321	0.285	GREAT
D10-D20	MF	0.159	0.175	0.167	RE-GOA
		0.039	0.5	0.073	GREAT
	BP	0.292	0.346	0.317	RE-GOA
		0.162	0.69	0.263	GREAT
	CC	NA	NA	NA	RE-GOA
		0.227	0.412	0.293	GREAT

(c)

Fig. S5. RE-GOA based differential ATAC-seq analysis provides comprehensive results. (a) Differential peak num between two time points. We use ATAC-seq data of RA-induced mESC cells at day 0, 2, 4, 10, 20 (mESC, D2, D4, D10, and D20), then filter the peaks and get differential peaks between two time points. (b) Distribution of number of peaks according to their distances to the nearest defined REs. Averagely, about 40% of peaks have their distances to the nearest REs less than 1 kb. (c) Comparison between the results of RE-GOA and GREAT. Taking results of gene set enrichment analysis of the differential expression genes in the same time period as golden standard, RE-GOA yields a higher f1-value in most of the time periods and GO parts.

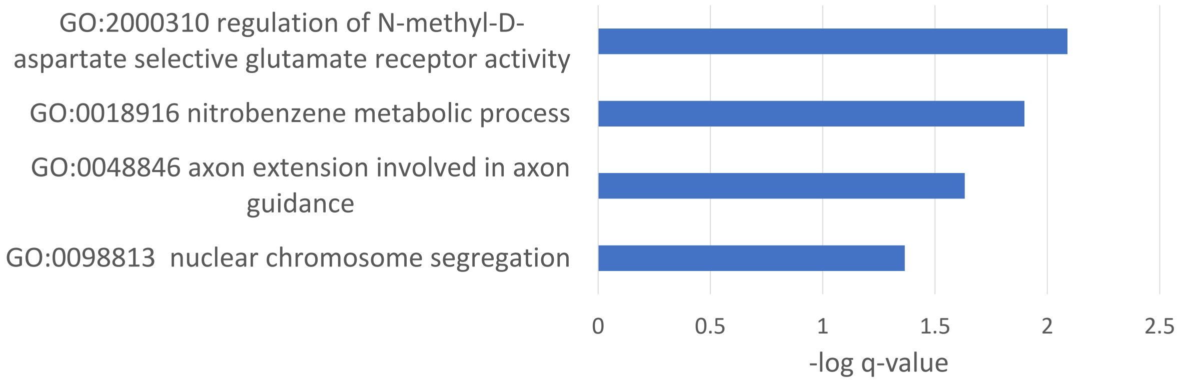

(a)

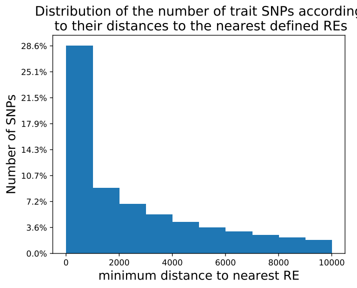

(b)

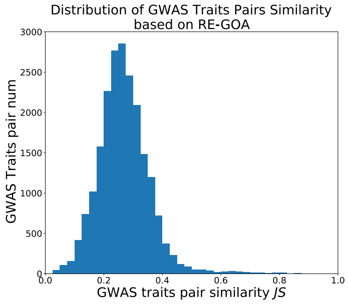

(c)

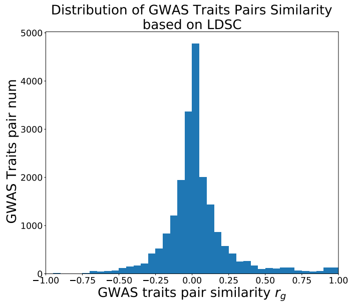

(d)

Fig. S6. RE-GOA reveals genetic correlation among phenotypes from their GWAS summary statistics data (a) Only 1 out of 4 terms enriched in GREAT analysis result of BMI SNPs is related with metabolic process (b) Distribution of the number of trait SNPs according to their distance to nearest RE defined. For 30% of the peaks, their distances to nearest REs are less than 1kb. (c) Distribution of the similarity of traits in pair based on RE-GOA. Most of trait pairs have a similarity between 0.2 to 0.4. (d) Distribution of the similarity of traits in pair based on LDSC. Most of trait pairs have a similarity around 0 based on LDSC.

The workflow of embedding of the constructed heterogeneous network is described in Algorithm S1.

Algorithm S1 Embedding of heterogeneous network constructed

Input: Networks: GRN, PPI, GO, GOA; $walklength$, $walknum$, $embeddingDim$, $iterize$
Output: $vectors$ ▷ $vectors$ representations for the nodes, including $vectors[T], vectors[F], vectors[G], vectors[R]$

function RandomWalk(GRN, PPI, GO, GOA, $walklength$, $walknum$)
    $paths = \emptyset$
    for G in $\{genes\ in\ GOA,\ PPI\ and\ GRN\}$ **do**
        for i in $\{1, 2, ..., walknum\}$ **do**
            randomly generate walks with defined metapaths ($GRFRG$, GTT, GTG, $GTTG$, GGG) beginning with gene G
            $paths = paths \cup \{walks\}$
        end for
    end for
    for F in $\{TFs\ in\ GRN\}$ **do**
        for i in $\{1, 2, ..., walknum\}$ **do**
            randomly generate walks with defined metapaths (FRF) beginning with TF F
            $paths = paths \cup \{walks\}$
        end for
    end for
    for R in $\{REs\ in\ GRN\}$ **do**
        $regR = \{G|R\ regulates\ G\ in\ GRN\}$
        for G in $regR$ **do**
            for T in $\{T|((G,T) \in GOA) \vee ((T', G) \in\ GOA\ \wedge T'\ is\ a\ descendant\ of\ T)\}$ **do**
                $paths = paths \cup \{[R,T]\})$ ▷ $[R,T]$ is chain generated followed the meta-path RT.
            end for
        end for
        for i in $\{1, 2, ..., walknum\}$ **do**
            randomly generate walks with defined metapaths ($RGGR$) beginning with RE R
            $paths = paths \cup \{walks\}$
        end for
    end for
    return $paths$
end function

$paths$ = RandomWalk(GRN, PPI, GO, GOA, $walklength$, $walknum$) ▷ first conduct random walk according to the metapaths defined
$vectors$ = word2vec($sentences = paths$, $size = embeddingDim$, $iter = iterize$)
▷ call the algorithm word2vec[1] to embed nodes (which are taken as words) in paths (which are taken as sentences in corpus) into low dimensional vectors.

---

[1]Mikolov, T. *et al.* (2013). Efficient Estimation of Word Representations in Vector Space. *ICLR*.

The algorithm for threshold selection is described in Algorithm S2.

Algorithm S2 Threshold Calculation

```
Input: GO, GOA, vectors
Output: Thres,                          ▷ Thres[T] is the threshold selected for term T
function CalSimG(GO, GOA, vectors)
    sim[G,T] = 0                        ▷ sim[G,T] stores the similarity of gene G and term T
    for G in {genes in GOA} do
        for T in {terms in GO} do
            s = cos sim(vectors[G]), vectors[T])      ▷ vectors generated in S1
            sim[G,T] = max(s, sim[G,T])
            for T' in {ancestors of T} do
                sim[G,T'] = max(s, sim[G,T'])
            end for
        end for
    end for
    return sim
end function

function CalThres(GOA, GO, sim)
    Thres[T] = 0                        ▷ Thres[T] stores the threshold selected for term T
    for T in {terms in GOA} do
        nowmaxf = 0
        standard = ∅
        for G in {genes in GOA} do
            if T in {T|((G,T) ∈ GOA) ∨ ((T',G) ∈ GOA ∧ T' is a descendant of T)} then
                standard.append(G)
            end if
        end for
        t = −1
        while t < 1 do
            t = t + δ                   ▷ δ is constable of a small amount
            predict = ∅
            for G in {genes in GOA} do
                if sim[G,T] > t then
                    predict.append(G)
                end if
            end for
            pre = |standard∩predict| / |predict|
            rec = |standard∩predict| / |standard|
            if (2*pre*rec)/(pre+rec) > nowmaxf then
                Thres[T] = t
                nowmaxf = (2*pre*rec)/(pre+rec)
            end if
        end while
    end for
    return Thres
end function

sim = CalSimG(GO, GOA, vectors)
Thres = CalThres(GOA, GO, sim)
```

The annotation algorithm is described in Algorithm S3

Algorithm S3 *RE-GOA* Generation

Input: GRN, GOA, GO, $vectors$, $Thres$
Output: $RE\text{-}GOA$ ▷ $RE\text{-}GOA[R]$ is the set of terms annotating RE R

```
function CalSimRE(GO, GRN, vectors)
    sim[R,T] = 0                          ▷ sim[R,T] stores the similarity of RE R and term T
    for R in {REs in GRN} do
        for T in {terms in GO} do
            s = cos sim(vectors[R], vectors[T])
            sim[R,T] = max(s, sim[R,T])
            for T' in {ancestors of T} do
                sim[R,T'] = max(s, sim[R,T'])
            end for
        end for
    end for
    return sim
end function

function RE-FunctionalAnnotation(GO, GOA, GRN, sim, Thres)
    GOA_raw[R] = ∅                        ▷ GOA_raw[R] stores the raw annotation
    for R in {REs in GRN} do
        regR = {G|R regulates G in GRN}
        for G in regR do
            GOA_raw[R] = GOA_raw[R] ∪ {T|((G,T) ∈ GOA) ∨ ((T',G) ∈ GOA ∧ T' is a descendant of T)}
        end for
    end for
    GOA_embed[R] = ∅
    RE-GOA[R] = ∅
    for R in {REs in GRN} do
        for T in {terms in GO} do
            if sim[R,T] > Thres[T] then
                GOA_embed[R] = GOA_embed[R] ∪ {T} ∪ {T'|T' is an ancestor of T}
            end if
        end for
        RE-GOA[R] = GOA_raw[R] ∩ GOA_embed[R]
    end for
    return RE-GOA
end function

sim = CalSimRE(GO,GRN,vectors)
RE-GOA = RE-FunctionalAnnotation(GO,GOA,GRN,sim,Thres)
```
